# Spontaneous Exercise-Related Coronary Artery Dissection among Young Patients Without Risk Factors or Atherosclerotic Disease

**DOI:** 10.36660/abc.20180446

**Published:** 2019-11

**Authors:** Pablo de Souza, Artur Haddad Herdy

**Affiliations:** 1 Instituto de Cardiologia de Santa Catarina (ICSC), São José, SC - Brazil; 2 Clínica Cardiosport de Prevenção e Reabilitação, Florianópolis, SC - Brazil

**Keywords:** Coronary Artery Disease, Myocardial Infarction, Death, Sudden, Cardiac, Young Adult, Risk Factors, Physical Exertion, Exercise, Diagnostic, Imaging/trends

## Abstract

Spontaneous coronary artery dissection (SCAD) is considered an often underdiagnosed acute coronary syndrome, with few cases described in literature. Its association with physical exercise among young patients without risk factors or atherosclerotic disease (CAD) is even rarer. For this reason, a study was conducted on the subject, describing the clinical conditions, conduct and evolution regarding the suspicion of spontaneous exercise-related coronary artery dissection in three young patients without risk factors or CAD. Clinical conditions varied, with predominant recurrent chest pain. Age range from 20 to 31 years. All patients underwent coronary angiography, which showed no CAD but suggested SCAD. Investigations concerning other causes of coronary obstruction were negative. The right coronary artery was affected in two cases, and the anterior descending artery was affected in one case. Only one of the three patients had recurrent events within five years from the primary event. Technological advances will enable increased dissection identification in acute coronary syndromes. Improving the knowledge about the related clinical conditions is necessary, as an attempt to provide warnings and improve the suspicion of spontaneous exercise-related coronary artery dissection among those who have symptoms of coronary insufficiency, thus reducing the frequent underdiagnosis. The best treatment and prognosis for this disease remains uncertain.

## Introduction

Often underdiagnosed in its history, spontaneous coronary artery dissection (SCAD) has been described as a rare etiology among acute coronary syndrome (ACS).^1-4^ Although it has been poorly studied, SCAD can result in significant morbidity, leading to ischemia and acute myocardial infarction (AMI), as well as ventricular arrhythmias and sudden cardiac death.^1-3,5-7^ SCAD cases are common in literature as isolated cases,^1,4-8^ and generally, this disease affects young women that had late diagnosis shown in a necropsy as registered in most publications. The first case described was in 1931.^1,2,9-12^

Recently, more cases of SCAD have been identified due to routine coronary angiography in ACS and technological advances in imaging.^1^

SCAD is associated with heterogeneous pathophysiological situations,^13,14^ as atherosclerotic disease (CAD), peripartum period, collagen diseases, genetic vasculopathies, cocaine and amphetamine abuse, anabolic and corticosteroid use, severe systemic hypertension, oral contraceptives, fibromuscular dysplasia, vasospasm and physical exercise.^1,2,13-22^

Physiologically, SCAD is defined as a separation of non-iatrogenic or traumatic coronary artery layers, creating a false lumen.^1-4,7^ This fragmentation can occur between the intimal and middle layers or between the middle and adventitial layers, with the formation of a intramural hematoma in the artery wall that compresses the artery lumen, reducing the anterograde blood flow with a subsequent ischemia or AMI.^1-4^ In some cases, a hematoma may communicate with the vessel lumen and a consequent thrombus formation at the endothelial lesion site. In addition, the possibility of endothelial injury caused by mechanical stress is speculated at some point in the vessel, leading to thrombosis at this site.^1,2,7-22^

There are few cases that associate SCAD with sports physical activities among young male patients without risk factors or CAD.^17-20^ In the present study, we describe three cases of ACS among young patients without risk factors and established or underdiagnosed CAD, where the main etiological suspicion of coronary artery flow obstruction was SCAD related to intense physical exercise, as concluded by the initial presentations, the complementary exams and the clinical evolution, considering the probable pathophysiological mechanism involved. The three cases presented were similar among each other.

## Methods

Three young male patients without risk factors and CAD who had symptoms of coronary insufficiency during or after intense physical exercise were evaluated. Outpatient or emergency investigations suggested SCAD.

The morbidity history of the patients was analyzed, excluding risk factors such as systemic arterial hypertension, smoking, use of drugs, anabolic steroids, ergogenic drugs, anorectic or illicit drugs, positive family history for coronary heart disease, cardiomyopathies or thrombotic disease. Cardiac catheterization (CATE) revealed a large amount of intracoronary thrombus, unrelated to CAD, suggesting SCAD as the main diagnostic hypothesis. We also researched and disregarded coagulation disorders after the occurrence of acute events. Table 1 presents the serial markers for thrombophilia, rheumatology diseases, inflamatory diseases, and connective tissue and negative serology researched in three patients.

**Table 1:** List of pathologies and procoagulant markers surveyed with negative results

Researched procoagulant pathologies and markers	
Leiden V Factor	Rheumatoid factor
Antithrombin III	ANF
Prothrombin Gene Mutation	Anti-Ro
Protein C and S	Anti-La
Activated protein C resistance	Anti DNA
Homocysteine	β2_ glycoprotein_ IgG and IgM
MTHFR Research	VDRL
Lupus anticoagulant	Anti-HIV
IgG and IgM anticardiolipin	Anti-HCV
pANCA	HBsAg
cANCA	Anti-HBc IgG and IgM

ANF: antinuclear factor; MTHRF: methylenetetrahydrofolate reductase; pANCA: perinuclear neutrophil anti-cytoplasm antibody; cANCA: cytoplasmic neutrophil anti-cytoplasmic antibody; HbsAg: hepatitis B detection antibody; anti-HCV: hepatitis C detection antibody; anti‑HIV: HIV detection antibody; anti-HBc: hepatitis B detection antibody.

### Case Descriptions

#### Case 1

Patient PS, male, 20 years old, amateur athlete, without risk factors for early CAD, does not use medication, anabolic, ergogenic, illicit or anorectic drugs. Negative family history for CAD, cardiomyopathy or thrombotic disease which began. Presents oppressive and burning chest pain, with irradiation to the left upper limb and chin, associated with nausea and insidious diaphoresis with six months of evolution. Symptoms appear after 30 min to 1h of high-intensity exercise, or sometimes only in the morning after strenuous physical activity and the night before, during a long period (up to 4h), once or twice a week.

**Figure 1:**
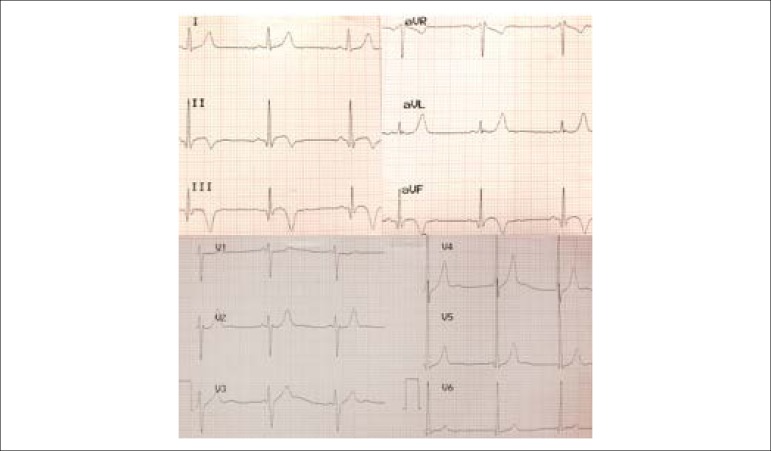
Emergency electrocardiogram during chest pain: Inferior subepicardial ischemia (T-wave inversion in DII DIII and AVF leads).

**Figure 2:**
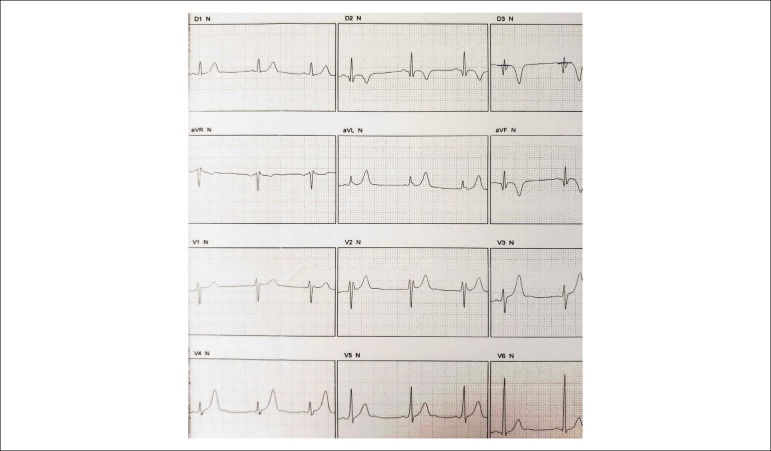
Resting and asymptomatic electrocardiogram: right bundle branch conduction disorder, V2 V3 and V4 peaked T waves, and persistent inverted T waves in inferior leads.

During six months, the patient sought emergency care several times. At times, during precordium pain episodes, the resting electrocardiogram (ECG) showed altered ventricular repolarization (ARV) in the lower wall with small Q waves and T wave inversion (Figure 1); when asymptomatic and at rest, it also presented a conduction disorder in the right branch and apical anterior septum T waves, considered at the time as changes that are compatible with a young individual that practices physical activities (Figure 2).

There were no changes in myocardial necrosis markers (MNM).

**Figure 3:**
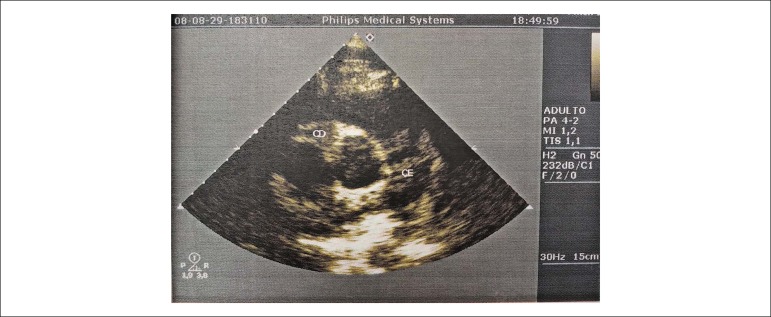
Evident dilation of right coronary artery on transthoracic echocardiogram at rest.

Continuing the investigation, a transthoracic echocardiogram (ECO-TT) was performed at the outpatient center, which showed a slight segmental disturbance in the left ventricular (LV) wall contractility at rest, with normal systolic function (LV ejection 64% fraction), as well as dilation in the right coronary artery (DCA), initially suggesting a coronary anomaly or fistula, and was advised to perform a CATE at the outpatient center (Figure 3).

The next morning, he sought emergency care once again presenting chest pain typically occurring when resting and with progressive chacteristics. There were 10 hours of evolution associated with diaphoresis.

During hospital admission, the patient presented regular general conditions, eupneic in ambient air, normalized, feverless and was anicteric. He presented high systolic blood pressure (220/140mmHg).

His weight was 78 kg; 1.78 m high and had a heart rate of 70 bpm.

There were no changes in cardiac and pulmonary auscultation.

Pulses were symmetrical with normal amplitudes and there was no peripheral edema.

Serial ECGs demonstrated the same changes as those described in the previous resting ECG during typical chest pain crises.

After morphine and nitrate analgesic measures, there was a partial response.

It evolved with a slight change in MNM, characterizing the condition as non-ST-segment acute myocardial infarction (STEMI).

**Figure 4 (A and B):**
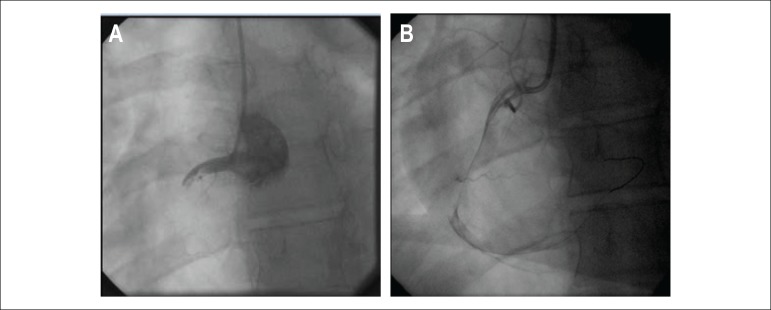
Cardiac catheterization: extensive thrombosis in the right coronary artery.

**Figure 5:**
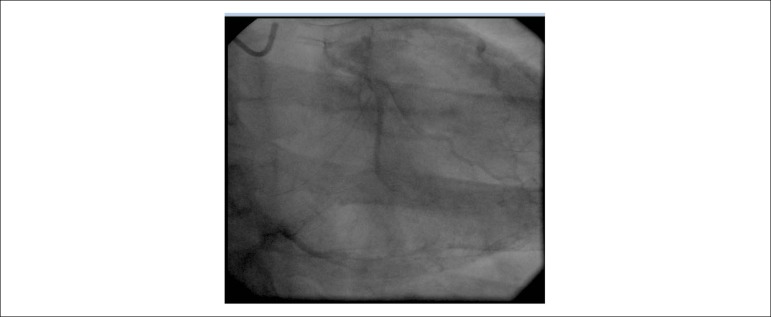
Cardiac catheterization: extensive thrombosis in the right coronary artery.

After risk stratification, the patient underwent CATE, which showed a large, large, a non-lesioned anterior descending artery (ADA) with major extensions and thick caliber that reached the middle third of the posterior interventricular sulcus, originating part of the posterior descending branch; non-dominant, non-injured circumflex artery (ACX) with a short extension and large caliber leading to a moderately important atrioventricular branch; Large SCAD with a large caliber apparently ectasized occluded with multiple thrombus images (Figure 4) and collateral circulation (CC) present with other uninjured arteries (Figure 5).

Primary coronary transluminal angioplasty (TCA) was performed using Pronto® Extraction Catheter and a Power Line® balloon catheter, with the removal of a large number of thrombi, a lesion with 100% occlusion began demonstrating some irregularities and a final TIMI 2 flow.

In the absence of CAD, other causes for coronary obstruction, such as thrombophilia, rheumatologic, inflammatory and connective tissue diseases, were investigated, but the results were negative.

P.S. was discharged one week after the event with no further symptoms. Simvastatin 40 mg was prescribed once a day to the patient; 50 mg atenolol was prescribed once per day, clopidogrel 75 mg once a day (as I was allergic to acetylsalicylic acid [AAS]); and enalapril 10 mg, 1 time/day. He remained using medication for two and a half years, and after that period discontinued use of the medication on his own.

He remained asymptomatic and practicing moderate-intensity exercise for another two and a half years.

After completing five years since the primary event, there was a recurrence of chest pain and associated symptoms, a few hours after intense and strenuous physical activity (jiu-jitsu and running).

When admitted, the patient was nauseated and had moderate to progressive chest pain, beginning 3 hours ago, in a retrosternal location, which improved after use of acetaminophen at home, and remained, on arrival at the emergency, continuous and poorly defined.

When performing a physical examination he was hypertensive, with a high level of diastolic blood pressure (170/140 mmHg).

An ECG was performed, which showed no acute changes.

**Figure 6:**
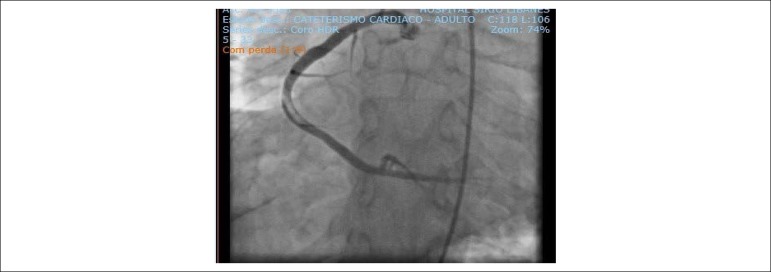
Cardiac catheterization: recurrence of thrombosis in the right coronary artery after five years.

**Figure 7:**
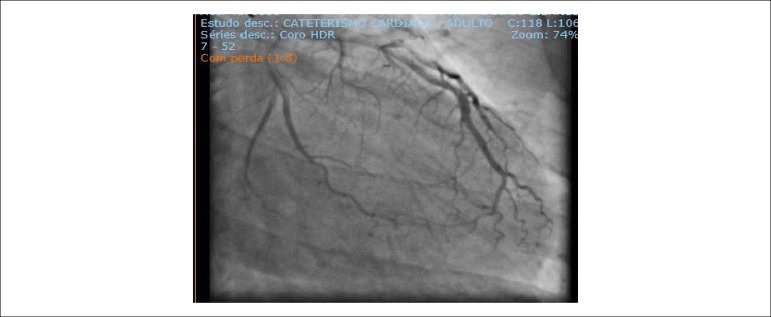
Cardiac catheterization: absence of collateral circulation from the anterior descending artery to the right coronary artery.

There was an increase in MNM, and he was diagnosed with a new STEMI and measures for ACS with consequent stratification to perform a new CATE that, once again, showed thrombosis in SCAD, without collateral circulation for SCAD from the left coronary (Figures 6 and 7).

He underwent TCA, with dilation and aspiration of intraluminal thrombi.

During hospitalization, atypical chest pain was felt two days after CATE. Coronary angiotomography (CT angiography) with zero calcium score was performed, which disregarded atherosclerotic lesions.

The patient was discharged 10 days after hospitalization and prescribed daily warfarin 5 mg; clopidogrel 75 mg and Ramipril 5 mg.

Other causes of coronary obstruction were disregarded in an in-hospital investigation.

Remains on medication and performs high intensity physical activities to this day; remains asymptomatic.

#### Case 2

Patient E.P.N, male, 29 years old, professional soccer athlete, without risk factors for early CAD, without previous use of anabolic, ergogenic, illicit or anorectic drugs. Negative family history for coronary heart disease, cardiomyopathy or thrombotic disease. He sought medical attention because of insidious retrosternal chest fatigue and discomfort evolving for a month, with strong intensity and short duration, related to intense physical efforts (soccer training) and relieved at rest.

He denied irradiation or associated symptoms, but presented progressive symptom worsening. The patient, who initially only had indefinite tiredness at the end of the match, ended up developing burning chest pain early in the training.

Upon physical examination, the patient was well overall, eupneic at ambient air, normal color, acyanotic, feverless and anicteric.

The patient weighed 79 kg; 1.79 m tall; with a heart rate of 60bpm; and blood pressure (BP) at 120/80 mmHg.

Chest examination showed an apical thrust and normal heart and lung sounds.

Pulses were symmetrical with normal amplitudes and there was no peripheral edema. Laboratory tests within normal limits.

**Figure 8 (A and B):**
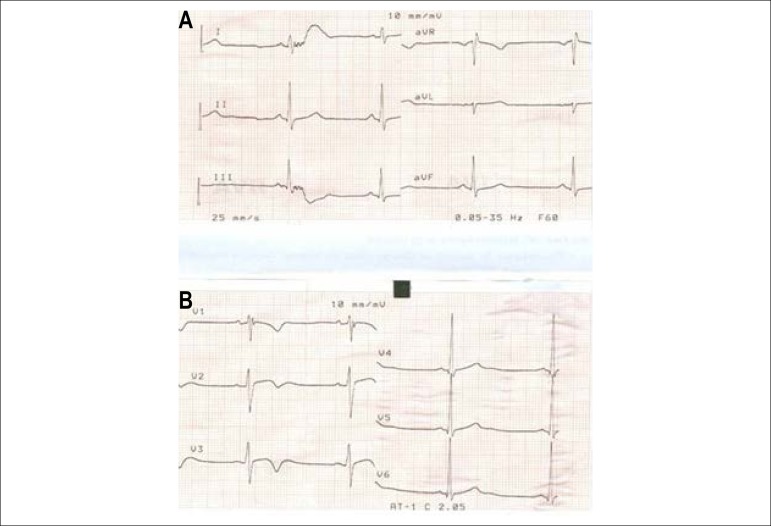
Electrocardiogram: T-wave inversion from V1 to V3.

Resting ECG evidenced ARV with anterior septum T-wave inversion (Figure 8).

Following the investigation, he underwent ergospirometric testing that showed no arrhythmias or electrocardiographic changes for myocardial ischemia, but there were symptoms of mild typical chest pain during the examination and abnormal findings concerning oxygen consumption (_VO2_) - 48.3mL/kg/min (the reference standard was 48.9); and the oxygen pulse was 21.1 ml 02/bpm (the reference standard was 19.9), with a plateau curve at the peak effort reached.

He then underwent transesophageal echocardiography, which showed normal wall thickness and dimensions, no septal defects and normal LV systo diastolic function, despite anteroapical hypokinesia.

The patient remained with anginal pain when an anatomical evaluation with CT angiography was necessary and showed proximal obstruction in the ADA which presented normal distal flow due to the receipt of collateral circulation in the right circumflex and coronary arteries.

**Figure 9:**
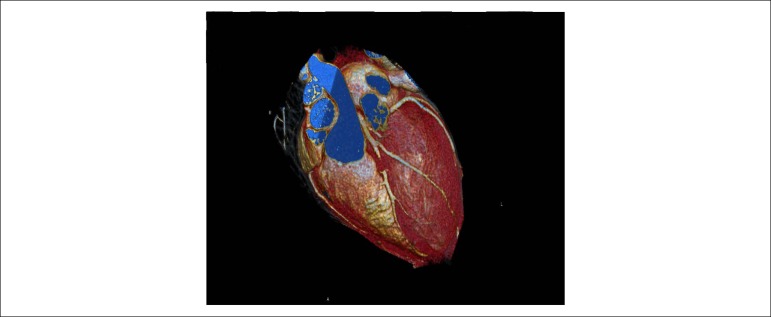
CT angiography: proximal obstruction of the anterior descending artery; normal distal flow.

**Figure 10:**
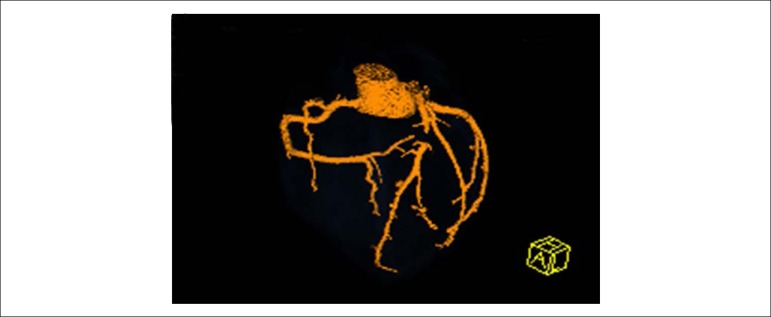
CT angiography: proximal obstruction of the anterior descending artery that receives collateral circulation of the right coronary artery and the circumflex coronary artery.

The examination disregarded coronary atherosclerosis (Figures 9 and 10). Thrombophilias, rheumatological, inflammatory and connective tissue diseases were also investigated; with negative results.

Due to the obstruction of the ADA, we investigated the repercussion of this lesion on ventricular function by myocardial scintigraphy, which showed transient hypocaptation in the anterior, apical and septal walls with great extent, reaching 28% of the LV.

Once the lesion was evidenced and its relevant repercussion was demonstrated, the patient was submitted to CATE, confirming the previous findings, in which the ADA TCA was performed with a pharmacological *stent* (Promus 4.0 × 2.8 mm), although the patient was aware of the possibility of distal embolization, resulting in a final TIMI 3 flow.

After the procedure, the patient remained asymptomatic receiving daily ASA, prasugrel and continued cardiac rehabilitation.

A control scintigraphy, after three months of CATE, demonstrated total reversal of myocardial ischemia.

Currently, the patient remains asymptomatic, performing outpatient follow-up, and practicing intense physical activity.

#### Case 3

Patient R.O.H, male, 31 years old, amateur soccer athlete (2 times/week), without risk factors for early CAD, without previous use of drugs, anabolic, ergogenic, illicit or anorectic drugs. Negative family history for coronary heart disease, cardiomyopathy or thrombotic disease. Sought medical guidance due to dyspnea and tiredness which started after practicing 1h of football.

Symptoms progressively worsened, progressing to moderate-intensity retrosternal chest pain and irradiation to the left upper limb, with a 2h course, with no other associated symptoms.

He reported a similar and single episode about a month earlier, in a similar situation, with spontaneous resolution after 2h feeling bad and having dyspnea.

On admission to hospital, he was in good general conditions, hypotensive, sweating, tolerating ambient air, normal color, acyanotic, feverless and anicteric.

He weighed 74 kg; was 1.69 m tall; and had a heart rate of 48 bpm; and BP 60/30 mmHg. BP maintenance was required, which increased rapidly after an infusion of 500 mL crystalloid (119/90 mmHg).

There were no changes in cardiac and pulmonary auscultation; extremities were not infiltrated.

On admission, the ECG showed ST-segment junctional rhythm in DII, DIII, aVF, V7, and V8 leads, and the was diagnosed with inferodorsal ST-segment elevation myocardial infarction.

Measurements for ACS were performed and referred to CATE, showing right dominance with severe proximal lesion (95%) in SCAD and a large amount of thrombi.

**Figure 11:**
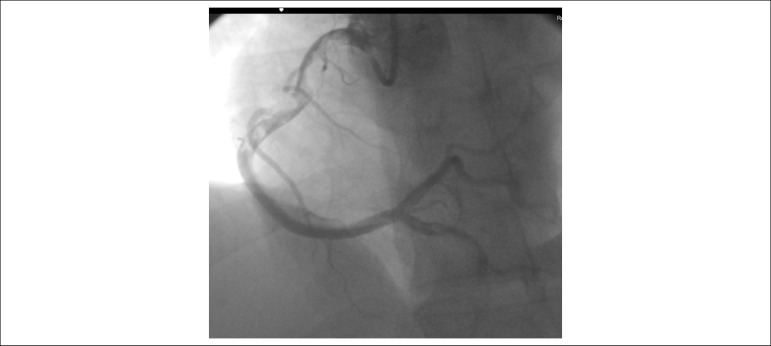
Cardiac catheterization: thrombosis in the right coronary artery.

**Figure 12:**
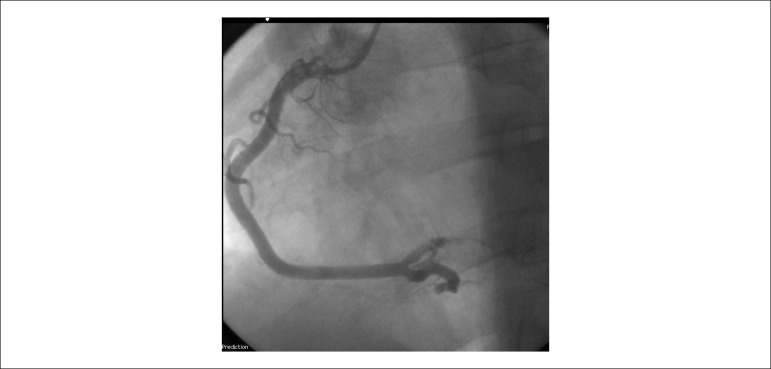
Cardiac catheterization: recanalized artery with distal thrombus migration.

Primary ATC performed for thrombus aspiration SCAD. Thrombi migrated to the distal portion of the ventricular and posterior descending arteries, and tirofiban was initiated (Figures 11 and 12).

He remained hospitalized for 4 days, with asymptomatic evolution, being discharged with a prescription of daily use of AAS 100 mg; clopidogrel 75 mg; atenolol 50 mg and rosuvastatin 10 mg.

Remains asymptomatic and has returned to football practice approximately 4 times/week.

## Discussion

Vigorous exercise is known to cause acute ischemia, but such events often occur in patients with established or underdiagnosed CAD.^19,23,24^

Reports of exercise-related SCAD in young patients without risk factors or CAD are rare in the literature.^17-20^ Most of the occurrences were described among young women related to the peripartum period, Marfan syndrome, oral contraceptive use, primary vascular diseases (vasculitis), or in patients with already diagnosed atherosclerosis or undiagnosed subclinical disease.^1-3,13,14,25,26^

Although some publications estimate the prevalence of ACSD between 23 and 36% in some populations (female),^2,13^ the actual prevalence of ACSD as the etiology of ACS in the general population remains uncertain.^1^

Recently, the *American Journal of Cardiology* published the analysis of the first major record of phenotypes involved in DEAC among the American population, using data from the *Nationwide Impatient Sample* (NIS). Data from 66,360 patients diagnosed with SCAD between January 2004 and September 2015 was evaluated.^14^ The average age was 61.3 ± 13.2 years and 44.2% were female. Depression was the most prevalent phenotype, which is why it was present in 5.15% of cases. Other causes frequently associated with SCAD, such as systemic vasculitis, Marfan syndrome, fibromuscular dysplasia, the use of steroids and corticosteroids, the abuse of cocaine or amphetamines, vasospasm, among others, were less prevalent (<1%). This analysis confirms that SCAD has a heterogeneous pathophysiological basis, in addition to the traditional causes of ACS. Smaller studies had previously described these conditions as a cause of SCAD. However, this was the first research to demonstrate that only genetic vasculopathies (Marfan and Ehlers-Danlos syndromes), fibromuscular dysplasia, the use of steroids and corticosteroids, migraine and some autoimmune and inflammatory conditions are more prevalent in ACSD through SCAD Than those not caused by spontaneous dissections.^14^ Fibromuscular dysplasia, for example, etiology frequently associated with SCAD in the literature and recently published its link to the genetic variant of the PHACTR1/DN1 allele rs9349379-A as the first identifiable genetic risk factor for SCAD,^2^ and it was prevalent in only 0, 16% of all cases analyzed.^14^

The age of the patients evaluated ranged from 20 to 31 years old, all male who practiced predominantly strong aerobic physical activity, one of them was a professional athlete and the other were amateurs.

While describing the case of a 54-year-old male patient with SCAD with no risk factors or CAD, Ellis et al. found 13 reports of exercise-related SCAD cases between 1995 and 2014^20^ Nine of these patients were male and the average age was 36 years, ranging from 17 to 53 years. Among these, seven cases (53.8%) were associated with aerobic activity and 5 cases (38.4%) were associated with anaerobic activity. Another case was also associated with severe emotional stress and anxiety. Most reported SCAD cases had risk factors or were diagnosed as atherosclerosis on the angiography, which cannot be considered true SCAD. The risk factors of each patient were analyzed: 30.7% were smokers, 30% had high cholesterol and 15.3% had a family history of ischemic heart disease. Obesity was identified as a risk factor in only 1 of these patients.^18^ Only one 25-year-old male, out of 13 patients,had SCAD without identifiable risk factors or atherosclerosis on the angiography.^20,27^

Apparently, when related exclusively to physical exercise, SCAD presents variable, recurrent and sometimes prolonged or subacute symptoms.

As reviewed by Ellis et al.^20^ and also in two of the cases described in this article, five of these patients presented themselves later after the dissection event, the longest was a patient with symptoms after a cycling *tour* and who suffered from angina for four months before seeking medical attention.^20,27,28^

In a literature review, Sherrid et al.^19^ also described 3 cases of exercise-related SCAD between 1965 and 1994, with varying clinical presentations.^19^ Two patients were female, 38 and 39 years old, respectively, and both had no risk factors. The first one died after manifesting pain in the arms followed by seizure, and an autopsy, an intermedial dissecting hematoma of the proximal portion of the ADA was found. At the time, the patient was shoveling snow (moderate exercise). The second patient presented anteroseptal and lateral AMI also when performing moderate physical activity (running > 40 km per week). During the CATE, a left lumen compressed by a false lumen secondary to dissection was evidenced. The other arteries were normal. Finally, the male patient presented cardiopulmonary arrest during high-intensity exercise (marathon training) at a ventricular fibrillation rate. During the CATE, a dissection on of TBI trifurcation was observed obstructing flow in the ADA. Both surviving patients were treated with myocardial revascularization surgery, showing no symptoms after the procedure.^19^

The case of DEAC in a 41-year-old woman on the ninth day of *IVF* therapy and without other risk factors was described by Balakrishnan et al.^18^ The patient who presented chest pain during high-intensity exercise (body pump) was diagnosed with STEMI by emergency resting ECG and underwent emergency coronary angiography. During CATE, a double-lumen signal, secondary to dissection, was observed in SCAD. Due to the instability of the condition during intra-artery contrast injection (by dissection propagation), a pharmacological *stent* and angioplasty was chosen. The patient had a good clinical course.^18^

The case of DEAC in a younger male patient was published by Kalaga et al., in 2007.^17^ This is a 17-year-old boy, captain of the school's football team, who felt a heavy burden in the chest during a friendly match of basketball. He had no modifiable risk factors, but his father had died at 38 due to a massive heart attack. The ECG showed anterior STEMI and CATE showed proximal ADA dissection with a large amount of thrombus and normal distal flow. Other arteries had no changes. He was treated conservatively with glycoprotein IIb/IIIa inhibitor and was discharged eight days after hospital admission. He performed a posterior physical stress test that did not demonstrate myocardial ischemia. He was advised not to participate in physical activities with intense competition, but released for moderate physical activity, since there was not, and currently is not, clear recommendation regarding the practice of competitive activities in patients with a history of physical activity-related DEAC.^17^

In the 3 cases described, no patient presented modifiable or non-modifiable risk factors for CAD.

In this sample, all presented chest pain or dyspnea, with recurrence of symptoms. These pictures suggest the formation of thrombi with varying degrees of stenosis, often with spontaneous resolutions and recurrence. These manifestations also suggest an endothelial mechanical lesion with thrombus formation at the injured endothelium site, as occurred in Case 1, with thrombus recurrence after years at the same site.

ADA is usually the most commonly affected artery in SCAD, and the incidence of SCAD ranges from 0.07% to 0.1% of patients undergoing coronary angiography.^1-16^

Although the literature suggests a better prognosis with conservative treatment (without *stenting*) and that ADA is the cause of most cases of SCAD, SCAD involvement was described in two of the three cases in this article. One of these two reported cases, both conservatively treated with balloon dilation and thrombus aspiration, had recurrence of thrombosis in the same artery five years later, in a similar situation, that is, after intense physical exercise, and the patient treated with ATC and a armacological stent remained asymptomatic, practicing moderate to intense physical activity until the present moment. Two patients were discharged with the prescription of dual antiplatelet therapy (DAPT), statin and a beta-blocker. One patient started using angiotensin-converting enzyme inhibitor (ACEI) and did not receive DAPT upon discharge due to being allergic to AAS.

The use of heparin and DAPT in DEAC is still controversial.^1^ The use of beta-blockers is recommended, and ACE inhibitors remain uncertain in these cases.^29^^.^^30^

Technological advances will enable more accurate diagnoses of SCAD among those with symptoms of coronary insufficiency or ACS. However, the pathophysiology of exercise-related SCAD is complex and still poorly understood, leading to conditions with variable and often subacute clinical presentations, which, in addition to the severity and therapeutic urgency, especially in an emergency setting, are often underdiagnosed.

The current diagnostic difficulty and the lack of studies directed to the specific treatment of this disease stimulate further studies and therapeutic proposals for exercise-related SCAD.

## Conclusion

Case reports such as these are extremely important, since better knowledge of the clinical conditions and presentation aims to increase the suspicion of exercise-related SCAD. Future studies directed to the diagnosis and treatment of this pathology are necessary. In addition, it is necessary to alert the medical community about this possible cause of ACS among chest pain conditions in male patients without risk factors and practicing physical activities.
